# The Impact of the Deterioration on Wood by Chlorine: An Experimental Study

**DOI:** 10.3390/ma16030969

**Published:** 2023-01-20

**Authors:** Rúben D. F. S. Costa, Marta L. S. Barbosa, Francisco J. G. Silva, Susana R. Sousa, Arnaldo G. Pinto, Vitor F. C. Sousa, Bruno O. Ferreira

**Affiliations:** 1 ISEP, Polytechnic of Porto, 4249-015 Porto, Portugal; 2 INEGI—Institute of Science and Innovation in Mechanical and Industrial Engineering, 4200-465 Porto, Portugal; 3 i3S—Instituto de Investigação e Inovação em Saúde, Universidade do Porto, 4200-135 Porto, Portugal; 4 INEB—Instituto de Engenharia Biomédica, Universidade do Porto, 4200-135 Porto, Portugal

**Keywords:** degradation, materials, wood, municipal facilities, chlorine deterioration

## Abstract

The use of disinfection and cleaning chemicals in several municipal facilities, such as swimming pools and drinking water treatment plants, causes the degradation of various types of wood, which leads to failures in equipment and the corresponding need for maintenance. This degradation creates added costs for municipalities, as well as the closure of certain facilities due to curative or preventive maintenance and, in many cases, public health issues, due to the water being contaminated with deteriorating products. Through a thorough study of the degradation effect on the products, more resistant materials can be found which are able to withstand these adversities and increase the lifespan of wood in regular contact with chemical agents. This is achievable by the determination of the cost-effectiveness of the substitute material to replace these components with alternative ones, with properties that better resist the deterioration effects promoted by aggressive environments. No studies have been found so far strictly focused on this matter. The objective of this study is to evaluate the degradation presented by two types of wood, beech and oak, which are exposed to the action of chlorine in municipal facilities. This degradation varies according to the chlorine content and the materials’ time of contact with the chemical agent, allowing the selection of new materials which will provide an extended lifetime of the components, reducing maintenance drastically, as well as costs for the facilities and the risk to public health. The performed experimental tests have shown that the oak wood has the best results regarding chlorine degradation resistance.

## 1. Introduction

Chlorine is a chemical element of the periodic table, belonging to group 17 (its atomic number), and is referred to by the symbol Cl. Although this element is an irritant gas at ambient temperature and atmospheric pressure, with a greenish-yellow colour, it is generally sold in steel cylinders under pressure, so it assumes the liquid form, transition which occurs at 6.8 atmospheres and 20 °C [1].

Despite both being used to disinfect water, chlorine (Cl) and sodium hypochlorite (NaClO) show some differences between them, such as their concentrations employed in the industry [2]. Chlorine can be obtained through the electrolysis of a sodium chloride (NaCl) solution. Initially, the chlorine in this solution is in gaseous form and, after decreasing its temperature and compressing it, turns into liquid [3]. Therefore, this element is employed for water treatment in liquid form, employed in swimming pools or for human consumption [4]. Additionally, chlorine is also used as a raw material in the production of some compounds such as dichloroethane, hydrochloric acid, hydrogen chloride and sodium hypochlorite [5,6,7,8], the last of which is the result of a reaction between chlorine and a dilute caustic-soda solution. It is mainly used in the production of disinfectants, in the liquid state, and having a chlorine content of 10% to 15% [9].

Wood is one of the oldest building materials [10]. This material is particularly valuable in comparison to the degrading conditions of most common materials when exposed to acids or a more aggressive environment, as it does not present such harmful degradation products. An example of this is that wood presents resistance to washing and is not subject to electrochemical corrosion since it is a non-electricity-conducting material [11,12,13]. In general, the use of this kind of material has always been highly questioned in moisture environments, mainly concerning its durability [14]. With technological advances and due to huge research, chemical substances capable of increasing the lifespan of wood have been discovered, as well as thermal modification and other modification processes. These can improve its desired properties, by increasing its safety, quality and durability, reduce costs due to possible reconstructions, and increase its versatility [15,16]. The wood quality increases when it is appropriately treated [17]. Its treatment increases resistance to certain aggressions, such as chemical reactions, hygrometric variations and mechanical solicitations [18,19]. Nevertheless, these treatments increase the cost of the material and do not always make it possible to achieve the properties shown by other families of materials.

In contact with certain chemicals, wood shows good results, even under severe conditions, since the chemicals enhance its properties [20]. In general, impermeable woods perform better with chemicals, presenting degradation mainly on their surface due to all the sap from the wood being removed, which is generally less harmful than metal corrosion products [21]. Wood is still a widely-used material for equipment in contact with chemical products and effluents, although its application has been decreasing with the use of synthetic polymers and other materials resistant to degradation, namely composite wood-based materials [22]. However, wood can be one of the most economic materials resistant to deterioration when prepared properly [23,24]. Due to the fact that, generally, studies focus on wood’s resistance to fungi or bacteria, this study intends to fill the gap concerning the chlorine resistance of different types of wood. Nitric acid, chlorine and sulphur dioxide are quite destructive to wood, attacking a component called lignin, causing surface defibration [25,26]. One way to avoid or minimize the influence of chemicals against the wood surface is to apply a protective coating. Several types of coatings exist, which can be made of various waxes, bitumen, chlorinated rubber, or chloride polyvinyl, among other materials [27,28]. Depending on the intended applications of this material, degradation manifests itself in various ways. In maritime or chemical conditions, the degradation manifests itself by defibration caused by the growth of salt crystals retained in the wetting and drying cycles, such as chlorine, nitric acid, sulphur dioxide and sulphites. These attacks are typically superficial in the case of treated woods, and deep with permeable wood species [29]. The chemical degradation of wood by metal corrosion products can be caused by four reasons [30]:

- poor construction;
- poor maintenance;
- unsuitable wood species (permeability); and/or
- inadequate durability of the metal fastener for the specific environment.

These reasons are explained due to the contact of the wood with corrosion products from metals. If the construction of certain facilities is not correctly done, as well as its maintenance, metal equipment may decrease its durability and corrode more easily, generating corrosion products which, in contact with wood, will negatively affect it.

These facts allow the electrolytes and the access to oxygen to promote a corrosive attack on metals. The chemical decomposition of wood by an alkaline medium occurs in cathodic areas (exposed metal and presence of oxygen). On the other hand, the softening and embrittlement of wood occurs in anodic areas (embedded metal and absence of oxygen), caused by the mineral acid from the hydrolysis of the corrosion product of soluble iron. Table 1 shows a list of woods with their degradation aggressiveness level and respective pH values [30].

Although the cases of degradation associated with woods are not very frequent, the possibility for this material to suffer decomposition, giving origin to deteriorating products, must be considered. Natural (untreated) wood can emit these products, generally composed of acetic acid, originating from the hydrolysis of organic substances, such as acetylated polysaccharides [31,32]. Despite small quantities of formic, propionic, and butyric acids being formed, the acetic acid is the major element responsible for the deteriorating action of wood [33].

In tests performed on wood in aqueous extracts, these showed a general correlation between quoted pH values and corrosivity of wood vapours. It was concluded that strongly acidic wood (pH < 4) is potentially dangerous, but the material is relatively safe if it has a pH > 5 [30]. Thermal treatments on wood are relatively dangerous to the environment and its life forms, including humans, since the acid in the vapour can be expelled, forming other types of vapours and accelerating hydrolysis. Volatile acid hardeners, such as hydrochloric acid in plywood glues, contribute to degrading vapours being expelled, as do varnishes and paints. It was also concluded that some conservatives may not affect the emission of degrading vapours from wood; however, some copper-based conservatives may reach enough leachable copper ions, which may lead to galvanic corrosion of other metals, mainly aluminium and steel [30]. Despite an intense search for studies focused on wood degradation due to contact with chemical products, mainly with chlorine, no studies have been found in this field. Thus, it is in the interest of the scientific community that this subject is now studied in detail given that some applications promote the contact of chlorine with wood artifacts, particularly swimming pool facilities and their surroundings.

However, other studies have been carried out regarding wood degradation in certain environments. Raberg et al. [34] studied the degradation of Scots pine and beech wood in four different environments in order to determine the influence of some wood preservatives. Images of the observed degradation were taken, which, combined with other previously-known data concerning rot type and fungal genus/species, allowed the evolution of knowledge and possible future approaches for testing new wood protection systems. Terrei et al. [35] studied the degradation of spruce wood under inert atmosphere (argon) and three different heat fluxes (38, 49 and 59 kW/m^2^). The insertion of micro-thermocouples allowed for analysing the evolution of temperature in depth in the wood, as well as the loss of mass resulting from the exposure of the wood to the environment selected for testing. The experimental results did not differ much from the results estimated by simulation, the difference being attributed to differences in thermal conductivity and specific heat considered for simulation. Elam and Bjordal [11] investigated wood degradation in urban soil-water systems over a long period of time, in order to characterize the behaviour of historic foundation piles. Further degradation was felt the deeper the wood was in the ground. The main cause of degradation was erosion. Microbial activity was considered as the main cause of the observed degradation. No relationship was detected between the presence of redox and oxygen, and the degradation observed in the wood. The same authors [36] carried out a comparative study of nine wooden foundation piles in historic buildings located in Gothenburg, correlating their behaviour with the usual characteristics of these woods, the environment in which they were inserted, and the service time. At the top of the piles, bacterial infection was detected in the center of the wood, which resulted in the cracking of the piles. Degradation is also influenced by the orientation of soil water flows. It was also possible to conclude that thicker piles are less affected by degradation than thinner piles. Yin et al. [21] studied the degradation of physicochemical properties and the differences in behaviour in terms of wettability of super hydrophobized birch and acetylated birch wood when exposed to UV radiation for a long time. The combined effect of surface modification and wood acetylation resulted in an improvement in wetting resistance, even after 600 h of UV exposure. Surface-modified wood samples were affected by a significant colour change after UV exposure. FTIR spectra showed that lignin was degraded both on unmodified wood surfaces and on wood surfaces enriched with silicone nanofilaments.

This work has the main objective of studying the degradation problems which arise because of the chlorine use in municipal facilities. In accordance, the sub-objectives are to identify these problems through the measurement of the components subject to degradation during operating time. Thus, two types of wood with the best intended properties have been selected (beech and oak). The immersion of these woods in different NaClO concentrations for different periods of time will allow us to determine the most suitable wood to be used in this kind of application. The objective of these immersion tests is to evaluate the performance of the different woods in contact with chlorine, and whether they are resistant to degradation or not, according to the exposure time and NaClO concentration.

**Figure 1:**
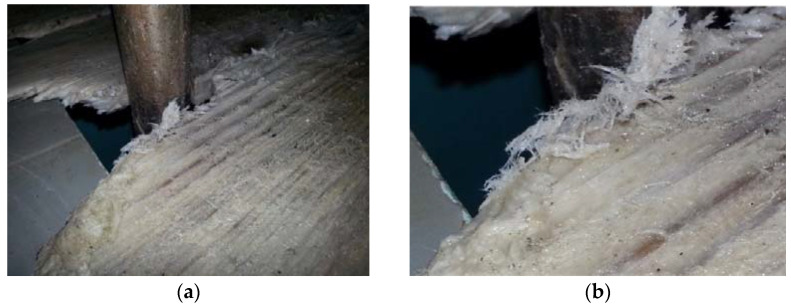
Degradation of the wooden cover of the drinking water treatment facility’s internal reservoir (**a**), and respective detail (**b**).

To identify the groups of wood which are most vulnerable to degradation in municipal facilities, such as swimming pools and drinking water treatment plants, the first step is to perform a survey of the degraded materials found. In municipal facilities, the chlorine-filled environment has a negative effect on its equipment, damaging various sorts of components (made of different types of material). Several cases of deterioration of wood were found (varying according to NaClO concentrations used), with instances such as the covers of the reservoirs, or part of a building structure, such as door frames, exhibiting significant damage. In most cases, if this deterioration had been properly considered in the design stage and certain construction details were considered, the deterioration effects could have been avoided or, at least, minimized. All these degrading problems, checked to date, show that even woods with higher costs and, thus, more resistant properties are not immune to degradation when they are not best suited to the purpose. An example of wood affected by a high-concentration chlorine environment is presented in Figure 1.

## 2. Materials and Methods

The set-out objectives can be achieved by recurring to the following steps:

- selecting two types of wood as the most likely used in equipment linked to municipal swimming pools facilities;
- determining the best conditions to perform and evaluate these types of wood under forced NaClO contact (immersion); and
- analysing the wood degradation in terms of visual aspect, colour change, mass change and main mechanical properties for different solutions and different immersion times.

### 2.1. Materials

Some of the direct consequences of this degradation are the reduction of the water quality level in the facilities, as well as the harm of the operators’ well-being in their workplace. This can happen, because the degraded wood has particles that can be dissolved in the water, if in contact with it, which can then pose risks to the operators’ health. As such, severe actions must be taken so these places are as secure as possible for everyone affected and to preserve the lifetime of the equipment. The choice of a material can be inappropriate, which may lead to serious consequences for the supplier as much as the customer, ranging from failure of the product to a huge increase in costs. The selection of materials involves a large variety of functional factors such as the project requirements, material properties that specify these requirements, costs, and manufacturing processes. For the study of a new process or improvement of any existing project, it is needed to select materials to be able to establish a processing relationship, structure, properties, performance and meet the requirements of the end consumer, and this is usually a difficult task. To prepare this selection, specific fields must be considered, such as knowing the chemical characteristics of the material, its atomic arrangement, atomic stacking factor, physical properties, and mechanical properties among other factors, considering the role to be played by the final product. Since this study focuses on the study of materials that are in contact with drinking water, it must consider a range of materials that will not harm water quality, while not posing a risk to public health nor hindering work employees due to excessive damage to the material. As such, the most important characteristics the selected material for these types of applications must have include resistance to degradation, wear and impact, the cost of the material and manufacturing process, the material’s ductility, weight, stiffness, and mechanical resistance. It must also be inert.

Considering all these factors, the selected woods to be tested in this work are beech and oak. These two types of wood are characterized by their high density and durability, as well as their good mechanical properties. Despite being considered difficult to dry and saw, due to their properties, these woods are easy to work regarding finishing operations, such as sanding and varnishing, which are fundamental processes to protect this material as much as possible against external attacks, such as chemical agents.

To carry out this work, samples with two different sets of dimensions were extracted from blocks, with the objective to evaluate dimensional influence on the degradation. The natural wood fibres were always kept aligned in the same direction, avoiding different behaviours caused by the natural wood’s anisotropy.

### 2.2. Methods

This experimental study was carried out by immersion in a stagnant solution, since it is a simple and efficient method. Generally, it is in this type of test that a greater degree of degradation is achieved in a short period of time, the latter varying depending on the used material and solution. This type of test is widely used when rapid responses are needed. Like any other type of accelerated test that allows the evaluation of the materials’ degradation, the ideal is always to evaluate the samples by comparison, to obtain better interpretations of the results.

Initially, all samples were weighed, while dry, to evaluate future gain or loss of weight. The objective of this analysis was to observe the possible chemical changes suffered by the materials, after their immersion for the stipulated periods of time.

For the test, five containers were prepared for each type of material containing 2%, 5%, 25%, 50% and 100% NaClO, concentrations in % (*v*/*v*). In addition, ten samples with small dimensions (30 mm × 20 mm) and four samples of large dimensions (140 mm × 20 mm) were made for the experiments. In each container, two small samples were placed and, in both the containers with 5% and 100% sodium hypochlorite, two large samples were added. Half the samples had a test time of three weeks, and the remaining ones were only withdrawn three months after being immersed in the solution. The variables for each type of wood were the dimensions of the samples (small or large), the chlorine concentration and the immersion time.

To carry out the preparation of the different NaClO solutions, sodium hypochlorite and distilled water with a pH of 6.1 were used. The experiment was performed at a temperature of approximately 21 °C, which was maintained throughout the test duration. Finally, the containers were closed and protected with a transparent film, in which small holes were made, to release possible gases produced between the materials and the solution, and to diminish the contact with possible impurities in the solution.

The step of analysing the results is always quite critical and requires a preliminary evaluation in the choice of the most appropriate methodology. There are several pieces of equipment to characterize morphology and composition, as well as calculation techniques to determine the intensity, rate, and speed of degradation. The different methodologies were complemented, and a combination of different techniques used to characterize the obtained results. In this work, the used methods were:

- Visual inspection: Method performed with the naked eye, allowing the samples to be observed before being withdrawn from the respective containers; at the exact moment they are removed; and after drying and cleaning. After this first evaluation, the samples were washed with distilled water. The washing was performed with moving water, avoiding possible mixtures of deteriorating products between the woods in this manner, since this step had as its main objective the removal of impurities found in the materials. The samples were then placed in a location at room temperature (about 25 °C) for two days, so they could dry without contact with other materials (sandpapers or other similar materials), thus avoiding any removal of the base material.
- Mass variation: For this step, a Denver Instruments APX-200 Precision Analytical Balance was used to calculate the mass variation of the samples after the respective experimental tests’ immersion time, considering the initial mass of each sample (in dry conditions) of the selected materials as a comparison.
- Scanning electron microscopy: A morphology analysis of the submerged samples was performed, using an FEI Quanta 400 FEG scanning electron microscope, equipped with an EDAX Genesys energy dispersive X-ray spectroscopy microanalysis system. Only the most degraded samples were chosen to be submitted to this analysis (100% NaClO concentration), for both the wood types (beech and oak), after being submerged for three weeks and three months.
- Tensile test: The tensile tests performed in this work were carried out using a universal SHIMADZU tensile testing machine, model Autograph AG-X 100 kN. As a first step, the samples were identified and marked, tracing their useful length limits (in this case, two points were marked between claws at 90 mm), so that the elongation of the material could be verified later. The specimen was then tightened in the gripper of the machine, and the program was set up for the tensile test, in which information about the type of the material was requested. In this evaluation, the large samples (140 mm × 20 mm) were used. The sample immersed in 100% NaClO had to be evaluated under different conditions than the other ones, namely with a distance between claws of 60 mm, to obtain a greater contact area of the test machine grips, unlike the other ones. This was necessary since the material was very pasty, causing the grips of the machine to slip even after decreasing the distance between them. When the material was clamped by the claws, the release of fluid absorbed by the material when it was immersed in NaClO solution was observed, despite the long drying time to which it was submitted, which explains its pasty texture. This shows that part of the lignin was substituted by NaClO, and it was difficult to dry the deep pores created by the sample’s exposure to the sodium hypochlorite. The tests were performed at room temperature, and the displacement velocities were kept constant, being 1 mm/min in accordance with the ASTM D-143 standard and also guaranteeing a satisfactory number of points for the evaluation analysis. At the end, the evaluated samples were compared to a standard sample of each material, and the breaking zone of each sample was analysed. The main objective of this study was to observe the mechanical behaviour of the immersed materials in two different solutions of sodium hypochlorite without any preparation of the sample and using significantly higher parameters when compared to the real situations used in municipal swimming pools and water treatment stations. The tensile tests were conducted on four samples of each immersed material in distinct solutions and for different periods of time, as well as a standard sample as reference, as shown in Table 2.

In this study, the properties were determined perpendicular to the fibre axis. Often, when wood accessories are purchased or constructed for a particular application, the importance given to this parameter is neglected, but it is of great importance. Taking this fact into account, it is considered preferable to carry out the study by always focusing on the lower resistance of the material and, therefore, ensuring a resistance and durability always superior to the study done.

The results in terms of mass and mechanical strength are the average values obtained regarding the number of samples tested, as previously noted. The standard deviation values reported were always below 5%, showing a good repeatability in all tests (mass and tensile tests).

## 3. Results and Discussion

### 3.1. Initial Samples’ Weight

At first, all the samples were weighed using an analytical scale, with an associated error of 0.0005 g. Table 3 shows the initial mass of each sample of the two selected types of wood, first with the small samples (30 mm × 20 mm) and then the large samples (140 mm × 20 mm).

When the samples were immersed in the solutions, nothing relevant happened; however, after a few days of immersion, the obtained results were different, both regarding the type of material studied and the concentration of sodium hypochlorite (NaClO) used.

### 3.2. Phenomena Observed after the Immersion of the Samples

#### 3.2.1. Two Days of Immersion

After two days of immersion in the NaClO solutions, effervescence occurred in all tested materials, being lower at lower NaClO concentrations and higher at higher values.

**Figure 2:**
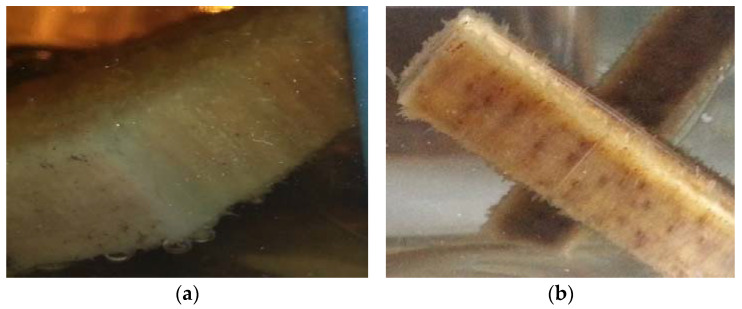
Degradation of beech (**a**) and oak (**b**) wood after two days of immersion.

In the samples of the two types of wood, there was a significant difference in the appearance of the material, in addition to a clearly different colouration in the solution, from yellow-green to brown tones, caused by the beginning of the material’s degradation, which is due to its defibration. Figure 2 shows the degradation of beech (a) and oak (b) in this time interval.

#### 3.2.2. Three Weeks of Immersion

After three weeks, the change in the solutions’ colouration compared with the one observed after two days expanded to darker shades, suggesting further degradation. By stirring the containers, the product caused by the degradation of the material was observed to be uniformly dispersed throughout the solution. This product presents an appearance of fine interlaced threads, caused by small fibres released by the material through defibration. All the solutions were analysed before the samples were taken from the containers, to verify the existence of sodium hypochlorite in them. The analyses revealed a total loss of chlorine in all solutions, caused by the fact that wood is an organic material, which reacted with the compound, absorbing it. After this period, there was no significant evaporation in any solution, but a slight negative variation in the container’s level occurred, due to the absorption of the solution by the wood samples, which, after being removed from the containers, showed a high degradation, as well as a significant decrease in their dimensions.

**Figure 3:**
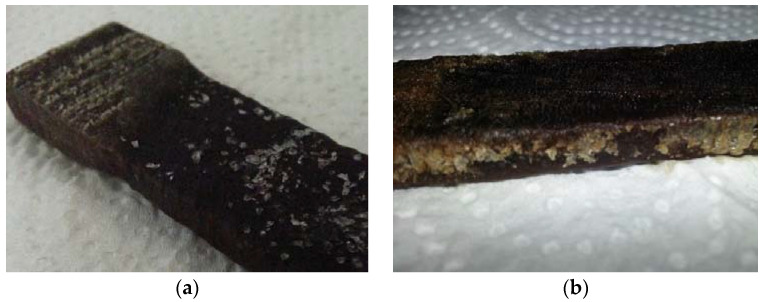
Dry samples of beech (**a**) and oak (**b**) after three weeks.

After being removed from their containers and washed, a visual evaluation of the samples was carried out, where it was possible to observe in detail the various induced levels of degradation (Figure 3).

In the dry samples, a high level of degradation was confirmed. The dimensions decreased when compared to the wet samples, and white salts appeared on their surface, caused by the contact of the materials with the sodium hypochlorite solution. In a tactile evaluation, both types of wood presented a fragile aspect.

#### 3.2.3. Three Months of Immersion

After three months of immersion, the phenomena visually verified are in general like those of the samples taken after three weeks, with the difference that the colouration in the solution has broadened to darker tones and there has been a more accentuated degradation of the wood, along with a more pronounced decrease in the size of the samples.

**Figure 4:**
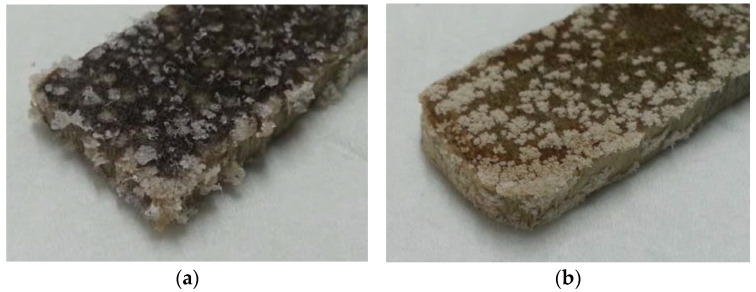
Dry samples of beech (**a**) and oak (**b**) after three months.

After being removed from the containers, the samples were washed and dried for two days. The result is shown in Figure 4.

In contrast with the three weeks of immersion, this time a very accentuated degradation of the samples was confirmed. It was also possible to verify the appearance of white salts in the samples’ surfaces in a much more significant amount than after three weeks. In a tactile evaluation, both types of wood presented a more fragile appearance of the sample.

Wood samples immersed in higher concentrations of sodium hypochlorite had greater difficulty in drying, presenting a darker colouration and higher concentration of salts on their surface. These presented significant moisture, due to wood being a highly hygroscopic material. Considering that, in a real situation, all wood components under study are often in contact with liquids and in a humid environment, the samples’ condition was considered acceptable to proceed with a new weighing, to determine the mass variation.

### 3.3. Mass Variation Analysis after Three Weeks and Three Months

Table 4 shows the mass variations obtained in each sample, expressed in grams, after three weeks of immersion.

Table 5 shows the mass variations obtained in each sample, expressed in grams, after three months of immersion.

#### 3.3.1. Mass Variation of the Small Samples

**Figure 5:**
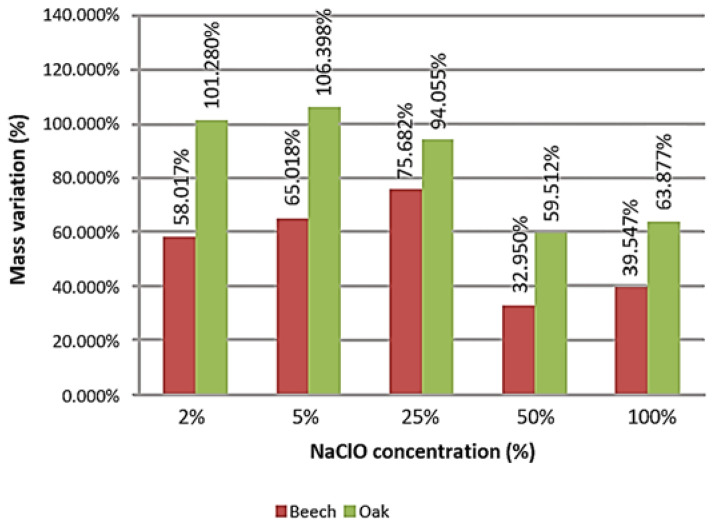
Mass variation of the small samples after three weeks of immersion.

Figure 5 represents the mass variation suffered by each smaller-sized sample of material. The value, expressed as a percentage, relates the variation in mass of each sample in relation to their initial mass, to evaluate which samples had a greater variation.

Both the beech and the oak increased in mass in all samples, the result being greater in the second case, caused by the absorption of liquid by the material. In the beech, there is a constant increase in mass between the samples containing 2%, 5% and 25% of NaClO concentration. Nevertheless, there is an abrupt decrease at 50% NaClO concentration, increasing again slightly at 100%. This phenomenon can be explained by the fact that the samples containing 50% and 100% NaClO suffered a more intense degradation, which caused a high loss of material and resulted in a lower mass variation in comparison with the other samples. The oak samples showed a similar behaviour. There was a linear increase in mass among the samples immersed in NaClO solutions of 2% and 5% NaClO, decreasing intensively in NaClO solutions of 25% and 50%. In summary, the oak samples did not lose as much mass as the beech ones for higher concentrations of NaClO, which shows a better resistance of the material.

**Figure 6:**
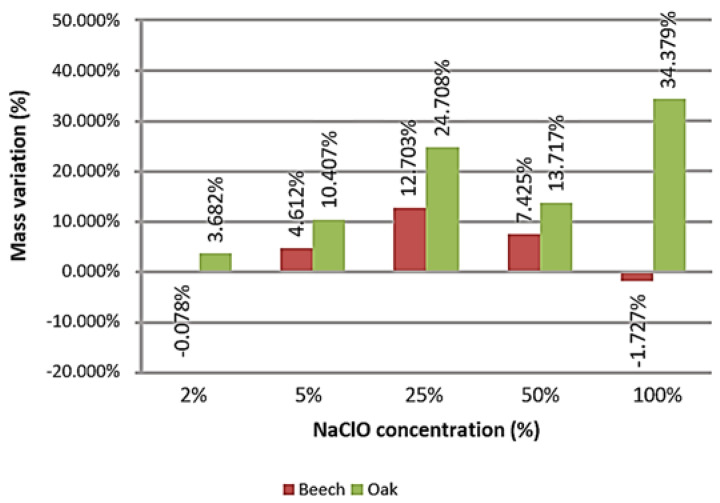
Mass variation of the small samples after three months of immersion.

The graph in Figure 6 represents the mass change experienced after three months in each small sample per material.

A constant increase in weight variation is noted among the beech samples containing 2%, 5% and 25% NaClO solutions of sodium hypochlorite. Nonetheless, there is an abrupt decrease in the NaClO solutions of 50% and 100%. The same happens for the oak, with the difference that for 100% concentration, there is an increase in mass variation. The samples immersed in 50% and 100% of NaClO presented a greater difficulty in drying. Almost all wood samples suffered mass increase; however, the beech ones presented a lower mass variation than the oak samples, when compared for the same concentrations. For 2% and 100% of sodium hypochlorite, the beech samples lost mass, indicating higher degradation of this material when compared to oak.

#### 3.3.2. Mass Variation of the Large Samples

**Figure 7:**
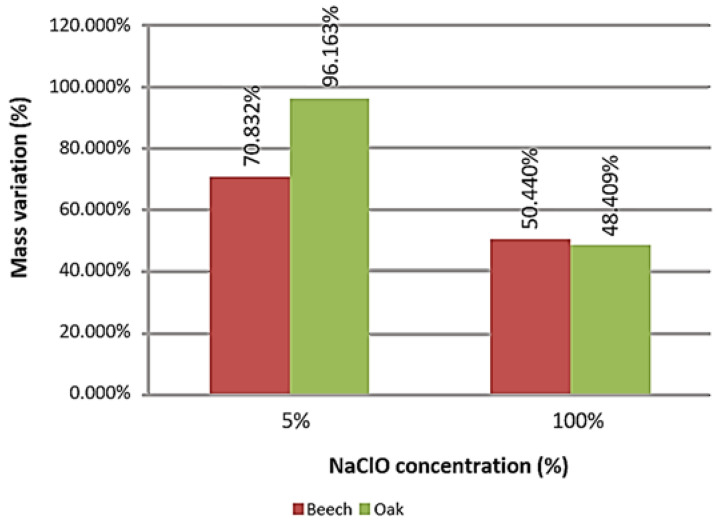
Mass variation of the large samples after three weeks of immersion.

The graph in Figure 7 represents the mass change experienced in the large samples per material.

Both samples, beech and oak, suffered mass increase. As in the small samples, it can be verified that there was a smaller variation of mass in the sodium hypochlorite concentration of 100%, due to the high material loss caused by deterioration. In this case, oak presents a greater variation in mass loss. At 5% NaClO, a significant mass difference between the two materials is observed, being higher in the oak sample. By contrast, at 100% NaClO, the differences in mass of the two kinds of wood reduced, being even slightly lower in the oak. This material had a worse reaction to contact with the sodium hypochlorite solutions, as it had a higher loss of mass.

**Figure 8:**
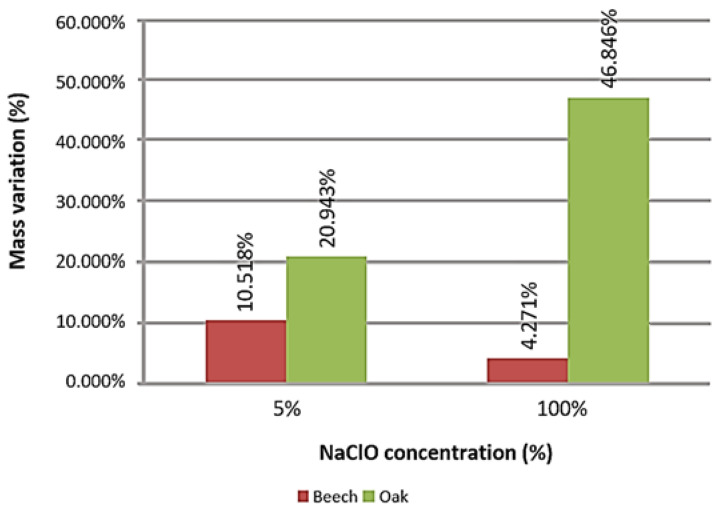
Mass variation of the large samples after three months of immersion.

The graph in Figure 8 represents the mass variation suffered after three months in each large sample, by material.

Both the beech and oak samples suffered mass increase. As in the small samples, the beech suffered a lower mass variation at a concentration of 100%, and the oak gained more mass. The higher variation is explained due to the mass of the fluid being absorbed by the material, remaining present during weighing. This is because of the high difficulty in drying the material, as it absorbed a solution with high levels of sodium hypochlorite.

### 3.4. Results Obtained by Electron Microscopy Analysis

Due to the high irregularity of the surface presented in the wood samples, it was not possible to perform any kind of analysis through optical microscopy, which is the reason why a morphology analysis of these samples was performed through scanning electron microscopy (SEM). As previously mentioned, only the samples immersed in 100% NaClO concentration were considered, as these suffered the largest amount of degradation.

#### 3.4.1. Beech wood

**Figure 9:**
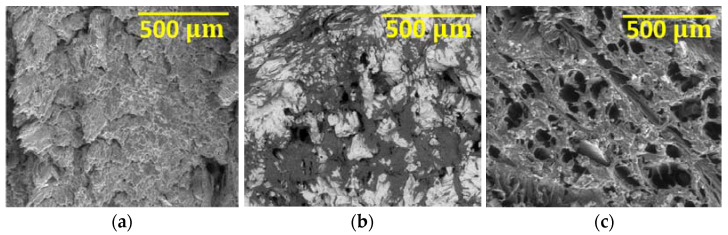
SEM image of the beech sample at a magnification of 200×: (**a**) before immersion, (**b**) after three weeks, (**c**) after three months.

Figure 9 represents the beech sample with a 200× magnification in three different situations: (a) without having been in any contact with the sodium hypochlorite solution, (b) after three weeks of immersion, and (c) after three months of immersion.

Figure 9a shows, on the surface, an apparently disorganized wood fibre structure, caused by the crushing of the fibres during the material cutting operation. In (b), it is possible to observe a high number of dark spots, which indicate the presence of pores, caused by the absorption of the solution by the material. On the other hand, the areas of white colouration represent the presence of salts coming from the sodium hypochlorite solution after drying. The rejection of these crystals from the wood’s interior originates the presence of holes and material loss, making the material more vulnerable to possible degradation attacks, since the fluid will have more contact with the material’s interior. Finally, in (c), the pores have increased in size, but the white coloured areas are more tenuous and less frequent, as there are fewer salts present on the wood samples, which were replaced by the pores, signal of the destruction of the wood fibres. Besides carbon and oxygen, as well as the previously-observed high chlorine and sodium content, calcium and aluminium were also present in these samples. These elements correspond to contaminations retained on the sample’s surface, caused during sample preparation and/or storage.

#### 3.4.2. Oak Wood

**Figure 10:**
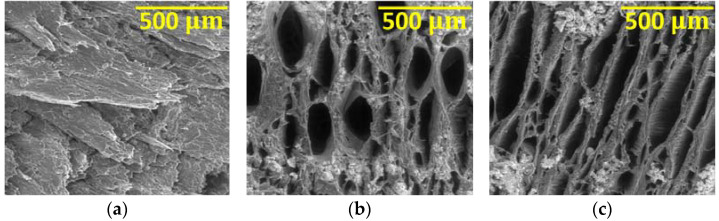
SEM image of the oak sample at a magnification of 200×: (**a**) before immersion, (**b**) after three weeks, (**c**) after three months.

Figure 10 represents the oak sample: (a) without having been in any contact with the sodium hypochlorite solution, (b) after three weeks, and (c) after three months.

In Figure 10a, where the unaltered aspect of this wood is observed, in contrast to the beech sample, the oak shows an organized structure of the wood fibres; however, there is also a crushed appearance of the sample’s surface. In (b) there is a greater number of dark spots and the presence of large pores, but fewer white coloured areas. The chemical analysis is like the one carried out on the beech samples, but the oak wood shows an aspect of greater degradation by comparison. Despite this, the oak sample presents a structure with higher fluid absorption capacity, due to the pore size observed, explaining the weight increase verified in the mass variation analysis. In (c), after three months of immersion, a remarkable number of pores due to the solution absorption by the material can be observed, as well as the presence of salts (white areas). Since the previous analysis, the wood suffered a great degradation, noticed by an increase in the pore size and by the destruction of lignin, leaving the fibres without a clear linkage.

### 3.5. Results Obtained in the Tensile Test

The last evaluation performed was based on uniaxial tensile tests.

The results observed in this analysis can also be influenced by the lack of pre-treating of the samples, which can occasionally influence their resistance. Nevertheless, the materials in service conditions also come into contact with chlorine without any kind of treatment, so this shows the usual conditions that the wood samples are subject to in municipal swimming pool applications until their total degradation.

#### 3.5.1. Beech Wood

**Figure 11:**
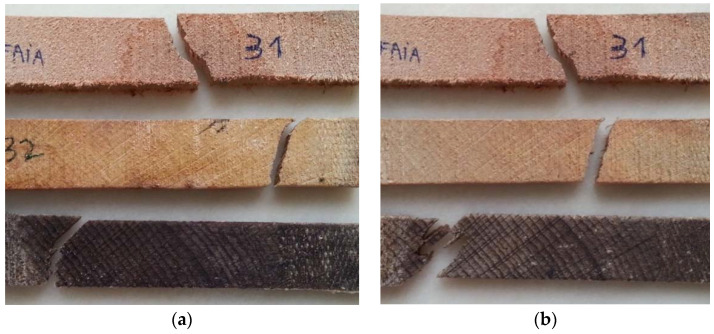
Beech samples submitted to the tensile test after three weeks (**a**) and three months (**b**).

Figure 11 shows the beech specimens after the tensile test, with the standard specimen at the top, immersed in 5% NaClO at the centre and in 100% NaClO at the bottom, after three weeks (a) and three months (b).

The samples immersed in 5% and 100% of NaClO rupture in approximately the same area, near the clamping of the test machine claws, for both cases. This fact can be justified by a significant difference in the cross-sectional area near the rupture zone, due to the degradation caused by the NaClO solution. On the contrary, the standard sample, which presents a uniform cross-sectional area throughout its length, suffered rupture in the central zone. In the 100% NaClO sample a chipped fracture was observed, typical in this type of material, being distinct from the other fractures. The samples after three months present a higher degradation aspect, with the wood starting to lose its colour.

**Figure 12:**
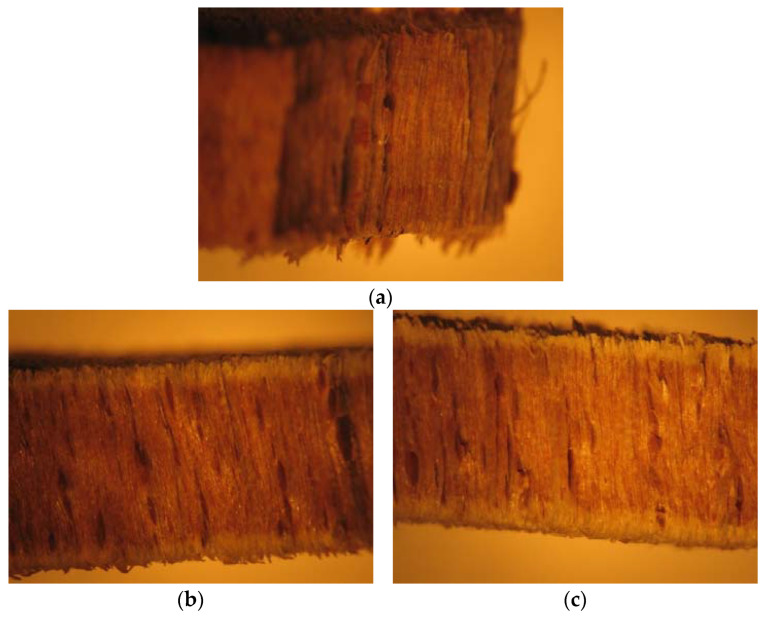

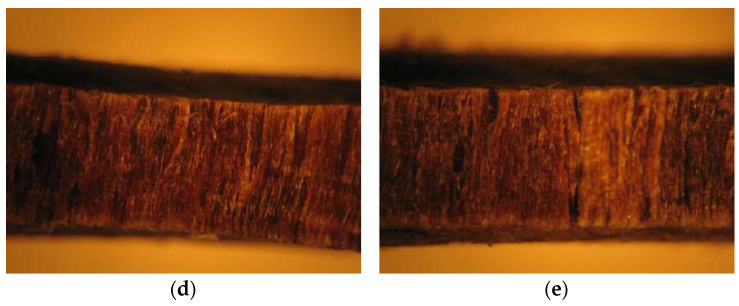
Fracture cross-section of the standard beech wood sample (**a**) and the samples immersed in 5% NaClO and 100% NaClO for three weeks (**b**,**d**) and three months (**c**,**e**).

Figure 12 presents the standard sample (a) and the beech wood samples immersed in 5% NaClO and 100% NaClO for three weeks (b and d) and three months (c and e).

From the images, it is possible to observe the rupture zone of the stressed samples, where a distinct fracture aspect among the three samples can be verified. The difference in section is caused by the removal of material caused by the contact with NaClO solution. Mainly in the sample immersed in 100% NaClO, it is possible to observe the high degradation suffered by the material when compared to the 5% NaClO-immersed and standard samples, a fact which had already been verified through electron microscopy. It also has a difference in colouration, indicating its deterioration, which led to a decrease in its dimensions, an irreversible process. Despite this, the internal structure of beech remains like the structure of the standard sample.

**Figure 13:**
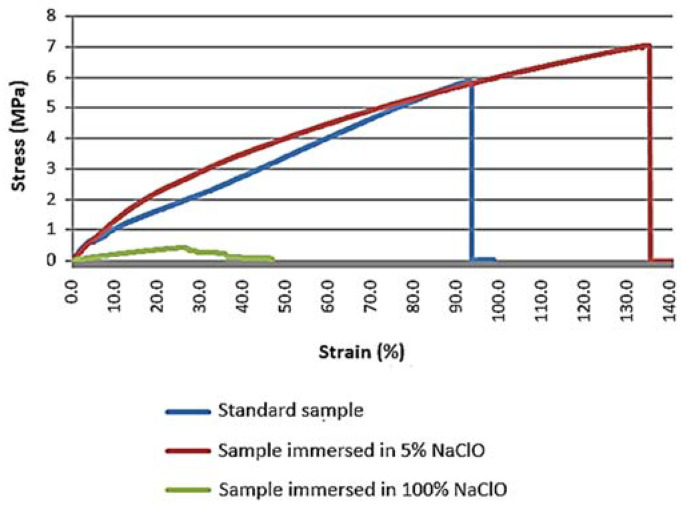
Stress-strain graph of the beech tensile test for the standard sample and samples immersed for three weeks in 5% and 100% NaClO.

The graph in Figure 13 shows the stress-strain curves for the different samples after three weeks.

The analysis of the graph shows that the mechanical properties of the sample immersed in 5% were affected by degradation, as there it presents a significantly greater elongation when compared to the standard sample. Nevertheless, no loss of mechanical resistance has been observed because the fibres remain acting as the main conveyor of the reaction to the tensile load imposed to the sample. The sample immersed in 100% NaClO is considered null for evaluation purposes, since it presents an inconclusive behaviour. In summary, the standard sample and the one immersed in 5% NaClO show similar behaviours, presenting themselves as fragile materials. Reaching the maximum strength, the material does not present brittleness.

**Figure 14:**
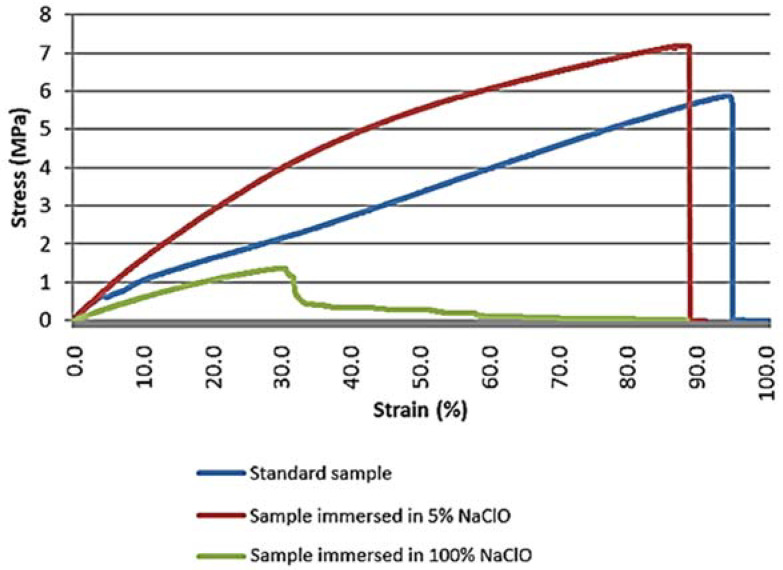
Stress-strain graph of the beech tensile test for the standard sample and samples immersed for three months in 5% and 100% NaClO.

On the other hand, after three months, after performing the test, it was verified that both the tensile strength and elongation presented significantly different values in relation to the standard sample, as presented in Figure 14.

This time, unlike the result after three weeks, the sample immersed in 5% NaClO has a relatively low elongation, even lower than the standard sample, having clear signs of the degradation suffered and of the solution absorbed by the wood after three months’ immersion. Once again, the sample immersed in 100% NaClO is considered null for evaluation purposes. By reaching the maximum strength, it can be verified that the material does not show any brittleness.

Table 6 shows the values reached by the material at the point of maximum strength in each sample after three weeks, as a function of its cross-sectional area.

Table 6 confirms the analysis made through the graph, and it can be verified that the sample immersed in 5% NaClO presents stress values close to those obtained by the standard sample. However, it proves the presence of higher elongations when compared to the standard sample. As already observed, the sample immersed in 100% NaClO did not present conclusive results and can therefore be considered as a total loss due to the damage caused by the NaClO solution.

These results agree with the theoretical study, where due to the degradation of the material, many open spaces were originated in the wood, as well as a subsequent increase in elongation when an external force is exerted [32].

Regarding the situation after three months, Table 7 shows the values reached by the material at the point of maximum strength in each sample, as a function of its cross-sectional area.

Table 7 supports the analysis made through the graph, and it can be verified that the sample immersed in 5% NaClO presented maximum stress values close to the values obtained by the standard sample, as the same that happens with the elongation. Once again, the 100% NaClO sample can be considered as a total loss. These samples presented a different behaviour from those removed after three weeks of immersion, since they presented less elongation when compared to the standard sample, reflected in the values obtained through the tensile test. This fact can be justified by the high level of degradation, which occurred due to a longer period of immersion for the samples.

#### 3.5.2. Oak Wood

**Figure 15:**
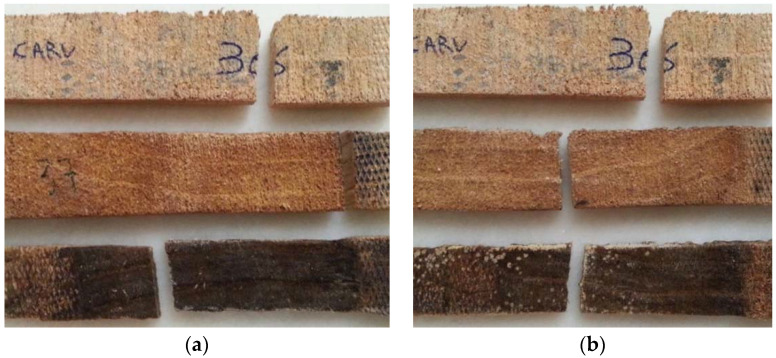
Oak samples submitted to the tensile test after three weeks (**a**) and three months (**b**).

Figure 15 shows the oak specimens after the respective tensile tests. At the top is the standard specimen, as a term of comparison, in the centre is the specimen immersed in 5% NaClO, and at the bottom the one immersed in 100% NaClO, after three weeks (a) and three months (b).

The sample immersed in 5% NaClO for three weeks has broken near the clamping zone of the claws of the testing machine, a form of rupture by pure tensile stress. Thus, there is a significant difference of the cross-section near the rupture zone. By contrast, the standard sample ruptured near the central zone, as did the 100% NaClO one and also every specimen after three months, which demonstrates that they present a uniform cross-sectional area dimension throughout their length. The samples immersed in 100% NaClO in both cases present a higher moisture content when compared to the other samples, proved by the darker colouration of the material. This fact is due to the high amount of fluid that remains inside the sample, not being naturally released as water. The retained fluid is sodium hypochlorite, since this phenomenon only occurs in samples immersed in concentrations of 100% NaClO. 

**Figure 16:**
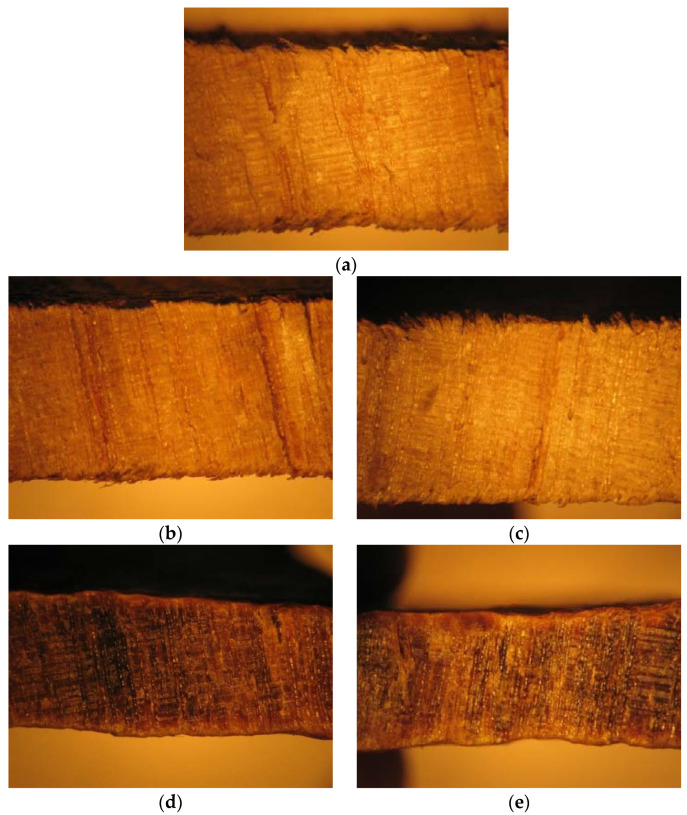
Fracture cross-section of the standard oak sample (**a**) and the samples immersed in 5% NaClO and 100% NaClO for three weeks (**b**,**d**) and three months (**c**,**e**).

Figure 16 shows the oak specimens after rupture.

From the images, it is possible to observe in detail the rupture zone of the samples, where a distinct fracture aspect among the three samples is visible. The fracture of the sample immersed in 5% NaClO is similar to the standard one, but in the sample immersed in 100% NaClO, it is possible to observe the aspect of intense degradation suffered by the material, alongside a decrease in its cross-section. The elongation of the immersed samples is caused by the enlargement of the pores of the material, also degrading the structure that resists the material, letting it elongate further. The internal structure of the oak remains like the structure of the standard sample. For the images after three months, there is clearly a more advanced state of degradation. Unlike the beech, which under these conditions presented a difference in colouring on the sample’s surface, indicating its deterioration, the internal structure of the oak remains like that of the standard sample.

**Figure 17:**
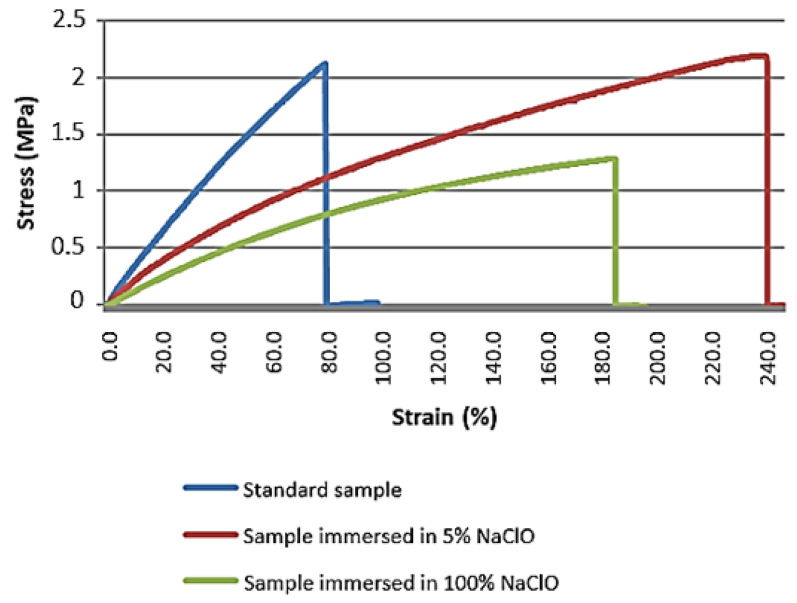
Stress-strain graph of the oak tensile test for the standard sample and samples immersed for three weeks in 5% and 100% NaClO.

After three weeks of immersion, the results obtained from the tensile test show that there are significant differences in the behaviour of the material, namely in terms of tensile strength and elongation, as can be seen in Figure 17.

The mechanical properties of the samples immersed in 5% and 100% NaClO were affected by degradation, immediately showing a significantly higher elongation of the sample immersed in 5% NaClO solution when compared to the standard sample. Even though the three samples show similar behaviour, they have different results, presenting themselves in general as a brittle material.

**Figure 18:**
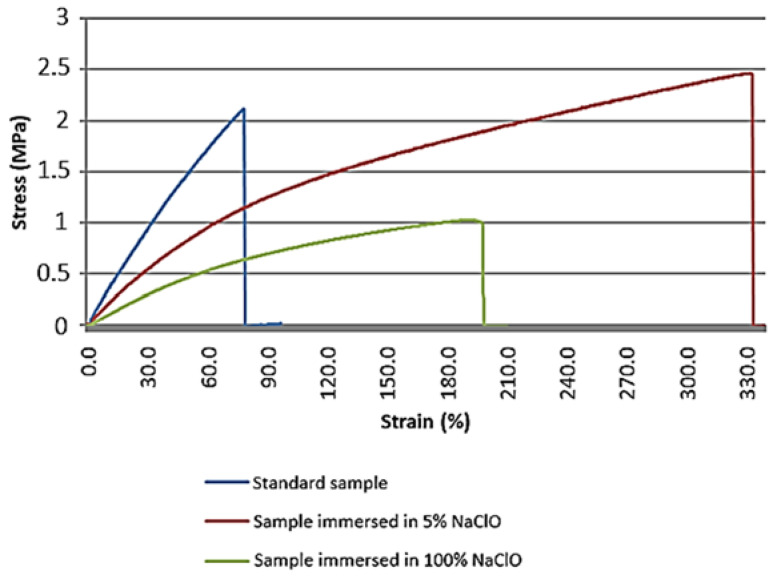
Stress-strain graph of the oak tensile test for the standard sample and samples immersed for three months in 5% and 100% NaClO.

On the other hand, after three months of immersion, Figure 18 shows the stress-strain graph for the test performed.

The graph proves the visual analysis carried out in the first stage, in which it is possible to see that the mechanical properties of the samples immersed in 5% and 100% NaClO were affected, with significantly higher elongations when compared to the standard one. The three specimens presented a brittle behaviour, not possessing strain.

Table 8 shows the maximum strength values reached by the material for each sample after three weeks as a function of its cross-sectional area.

The sample immersed in 5% NaClO presents maximum stress values close to the values obtained by the standard sample. As expected, the sample immersed in 100% NaClO showed lower maximum stress values than the other samples, as well as a stretching between the standard sample and the sample immersed in 5% NaClO, meaning greater degradation when compared to the latter.

Table 9 shows the values reached at the point of maximum strength in each specimen after three months as a function of its cross-sectional area.

The results are in line with those obtained for three weeks of immersion, having shown a behaviour within expectations—that is, the samples immersed in NaClO solution showed higher elongation results when compared to the standard sample, caused by the contact with the solution. Indeed, the solution increases the ductility of the wood structure and slightly improves the mechanical resistance in a consistent way, for immersion in solution of 5% NaClO. The same is not true for 100% NaClO because the lignin is seriously affected by the solution, leaving the main fibres not properly supported, rupturing easily when subjected to tensile tests.

The degradation of wood has been studied with some regularity, due to the importance of wood for the construction sector in some countries that have vast natural resources in this field and whose population has a strong environmental education, privileging natural resources and environmental sustainability. However, studies focus much more frequently on the resistance shown by different types of wood to fungi or bacterial activity, which have a harmful effect on wood preservation. Despite a thorough search, it was not possible to find any paper that studied the effect of the degradation caused by chlorine on any type of wood. This reinforces the innovative character of this study.

## 4. Conclusions

The study of the degradation caused by chlorine in different wood types has never been reported before in scientific studies. Thus, this work intends to fulfil this gap. The evaluations were carried out without any wood preparation, having as their objective the study of two kinds of wood usually used in municipal swimming pool equipment, being subjected to direct contact with various solutions of NaClO, trying to replicate common conditions that generally occur in real situations. Amongst the woods, a natural material, beech and oak were chosen.

Most studies were focused on 5% and 100% concentrations, since drinking water and water for swimming pools have a very low concentration (less than 5%), while the sodium hypochlorite feed pipes are in direct contact with its maximum concentration, as well as reservoirs, distributors, etc.

Beech gains resistance and elongation for a concentration of 5% NaClO, but the results were null for concentrations of 100% NaClO. Indeed, immersion in 5% NaClO solution shows a degradation in terms of wood colour, but the drying operation is relatively easy to perform, and the lignin and fibres are not degraded enough to induce losses in terms of ductility or mechanical resistance. In fact, for immersion in 5% NaClO, samples gained higher ductility and mechanical strength because the apparent initial brittleness exhibited by wood samples is softened by the immersion, clearly improving the strain and slightly increasing the mechanical strength. However, this is not true for immersions in 100% NaClO, because the internal structure of the wood is clearly affected and the linkage promoted by lignin among the natural fibres of the wood is clearly broken, inducing relevant loss of mass in the samples and removing the cohesive resistance that wood usually offers as a “natural composite” material. Moreover, maybe due to a higher viscosity of the 100% NaClO solution, as well as the more pronounced cavities generated in the samples, some solution seems impossible to remove from the inner part of the samples, which also conditions the mechanical behaviour, meaning it becomes very difficult to perform tensile tests in these samples. Hence, these results cannot be considered due to a permanent slipping of the samples between the claws of the tensile machine. The behaviour of the beech samples is also degrading over immersion time for the same type of solution. Thus, tests after three months presented clearly worse results than the ones performed over three weeks. Therefore, beech is affected both by NaClO concentration and duration of the immersion operation.

Oak, on the other hand, maintained a stable behaviour over time, with no accentuated degradation between samples with three weeks and three months of immersion. However, it lost mechanical strength for 100% of NaClO, which proves the poor capacity of this material in direct contact with chlorine. Mechanical strength was preserved for 5% concentration, also gaining elongation.

Finally, it can be concluded that woods have increased difficulties in resisting degradation caused by direct contact with NaClO, and should only be used in circumstances with low concentrations, i.e., only in contact with the chlorine atmosphere and not constant liquid media. Among the two types of wood studied, oak showed the best overall results in the conducted tests and should be the material of choice for the cases under study.

## Figures and Tables

**Table 1:** Degradation level of different woods under typical pH values [30].

Wood Degradation		
Wood	Degradation Level	Typical pH Values
Oak	High	3.35–3.9
Chestnut	High	3.4–3.65
Beech	Moderate	3.85–4.2
Birch	Moderate	4.85–5.35
Teak	Moderate	4.65–5.45
Cedar	Moderate	3.45
Pine	Low	5.2–8.8
Mahogany	Low	5.1–6.65
Walnut	Low	4.4–5.2

**Table 2:** Characteristics of the samples used for each material, regarding the concentration of NaClO and their immersion time.

Characteristics of the Samples Used for Each Material		
Quantity	NaClO Concentration	Immersion Time
1 (Standard)	0%	0 Days
1	5%	3 Weeks
1	100%	3 Weeks
1	5%	3 Months
1	100%	3 Months

**Table 3:** Initial mass of the samples regarding each test condition.

Mass of the Small Samples (g)										
Concentration	2%		5%		25%		50%		100%	
Duration	3 week	3 mon.	3 week	3 mon.	3 week	3 mon.	3 week	3 mon.	3 week	3 mon.
Beech	3.970	4.124	3.546	3.725	3.929	3.598	3.796	3.305	3.618	3.683
Oak	3.055	2.995	2.846	3.037	3.154	3.063	2.928	2.860	3.011	3.002
Mass of the large samples (g)										
Concentration	2%		5%		25%		50%		100%	
Duration	3 week	3 mon.	3 week	3 mon.	3 week	3 mon.	3 week	3 mon.	3 week	3 mon.
Beech	-	-	14.515	14.336	-	-	-	-	14.021	14.637
Oak	-	-	14.151	13.200	-	-	-	-	12.292	12.702

**Table 4:** Mass of the samples after three weeks of immersion.

Mass Variation of the Small Samples (g)		
NaClO Solution (%)	Beech	Oak
2	+2.303	+3.094
5	+2.306	+3.028
25	+2.974	+2.966
50	+1.251	+1.742
100	+1.431	+1.923
Mass variation of the large samples (g)		
NaClO Solution (%)	Beech	Oak
5	+10.281	+13.608
100	+7.072	+5.951

**Table 5:** Mass of the samples after three months of immersion.

Mass Variation of the Small Samples (g)		
NaClO Solution (%)	Beech	Oak
2	−0.003	+0.110
5	+0.172	+0.316
25	+0.457	+0.757
50	+0.245	+0.392
100	−0.064	+1.032
Mass variation of the large samples (g)		
NaClO Solution (%)	Beech	Oak
5	+1.509	+2.764
100	+0.779	+5.950

**Table 6:** Values achieved by beech at maximum force during tensile testing of the standard sample and samples immersed for three weeks in 5% and 100% NaClO.

Values Achieved by the Material at Maximum Force				
Sample	F_max_ (N)	Area (mm^2^)	σ_max_ (MPa)	Strain (mm)
Standard	939.10	160	5.86	2.84
5% NaClO	1127.51	160	7.04	4.12
100% NaClO	66.61	160	0.41	0.79

**Table 7:** Values achieved by beech at maximum force during tensile testing of the standard sample and samples immersed for three months in 5% and 100% NaClO.

Values Achieved by the Material at Maximum Force				
Sample	F_max_ (N)	Area (mm^2^)	σ_max_ (MPa)	Strain (mm)
Standard	939.010	160	5.86	2.84
5% NaClO	1147.99	160	7.18	2.68
100% NaClO	216.80	160	1.35	0.90

**Table 8:** Values achieved by oak at maximum force during tensile testing of the standard sample and samples immersed for three weeks in 5% and 100% NaClO.

Values Achieved by the Material at Maximum Force				
Sample	F_max_ (N)	Area (mm^2^)	σ_max_ (MPa)	Strain (mm)
Standard	339.02	160	2.11	0.68
5% NaClO	351.19	160	2.19	2.02
100% NaClO	205.83	160	1.28	1.58

**Table 9:** Values achieved by oak at maximum force during tensile testing of the standard sample and samples immersed for three months in 5% and 100% NaClO.

Values Achieved by the Material at Maximum Force				
Sample	F_max_ (N)	Area (mm^2^)	σ_max_ (MPa)	Strain (mm)
Standard	339.02	160	2.11	0.68
5% NaClO	394.19	160	2.46	2.86
100% NaClO	164.48	160	1.02	1.65

## Data Availability

Not applicable.
